# CHIP: a clonal odyssey of the bone marrow niche

**DOI:** 10.1172/JCI180068

**Published:** 2024-08-01

**Authors:** Wolfgang E. Schleicher, Bridget Hoag, Marco De Dominici, James DeGregori, Eric M. Pietras

**Affiliations:** 1 Division of Hematology, Department of Medicine, and; 2 Department of Biochemistry and Molecular Genetics, University of Colorado Anschutz Medical Campus, Aurora, Colorado, USA.

## Abstract

Clonal hematopoiesis of indeterminate potential (CHIP) is characterized by the selective expansion of hematopoietic stem and progenitor cells (HSPCs) carrying somatic mutations. While CHIP is typically asymptomatic, it has garnered substantial attention due to its association with the pathogenesis of multiple disease conditions, including cardiovascular disease (CVD) and hematological malignancies. In this Review, we will discuss seminal and recent studies that have advanced our understanding of mechanisms that drive selection for mutant HSPCs in the BM niche. Next, we will address recent studies evaluating potential relationships between the clonal dynamics of CHIP and hematopoietic development across the lifespan. Next, we will examine the roles of systemic factors that can influence hematopoietic stem cell (HSC) fitness, including inflammation, and exposures to cytotoxic agents in driving selection for CHIP clones. Furthermore, we will consider how — through their impact on the BM niche — lifestyle factors, including diet, exercise, and psychosocial stressors, might contribute to the process of somatic evolution in the BM that culminates in CHIP. Finally, we will review the role of old age as a major driver of selection in CHIP.

## Introduction: CHIP as an epic journey

Like the lengthy epic tales of the Greek poet Homer, clonal hematopoiesis of indeterminate potential (CHIP) is postulated to be the culmination of years, if not a lifetime, of clonal selection for mutant hematopoietic stem and progenitor cells (HSPCs) in the BM microenvironment. The most common CHIP mutations include loss-of-function mutations in epigenetic modifiers (*DNMT3A*, *TET2*, and ASXL1), splicing factor (SF) genes (*U2AF1*, *SRSF2*, *SF3B1*), and DNA damage–response (DDR) genes (*PPM1D* and *TP53*) as well as gain-of-function mutations in JAK2, among others (1–4). Although CHIP is defined by the absence of hematologic malignancy, such as myeloproliferative neoplasms (MPN), myelodysplastic syndrome (MDS), or acute myeloid leukemia (AML) (5), its association with myriad disease pathologies underscores its importance in contemporary hematology research. CHIP is strongly associated with older age, with around 15% of individuals over 75 exhibiting overrepresentation of CHIP mutations (1). CHIP is likewise associated with an increased risk for several aging-associated diseases, including solid cancers, heme malignancies, a variety of heart and lung conditions, and all-cause mortality (1, 6–14). Given the proportion of CHIP-associated diseases and diversity of mutations that drive CHIP, a key focus of the field has been understanding both intrinsic advantages of HSPCs harboring CHIP mutations and extrinsic BM niche changes with age, environmental exposures, infection, and other lifestyle factors that facilitate mutant HSPC competitiveness and expansion (15).

**Figure 1:**
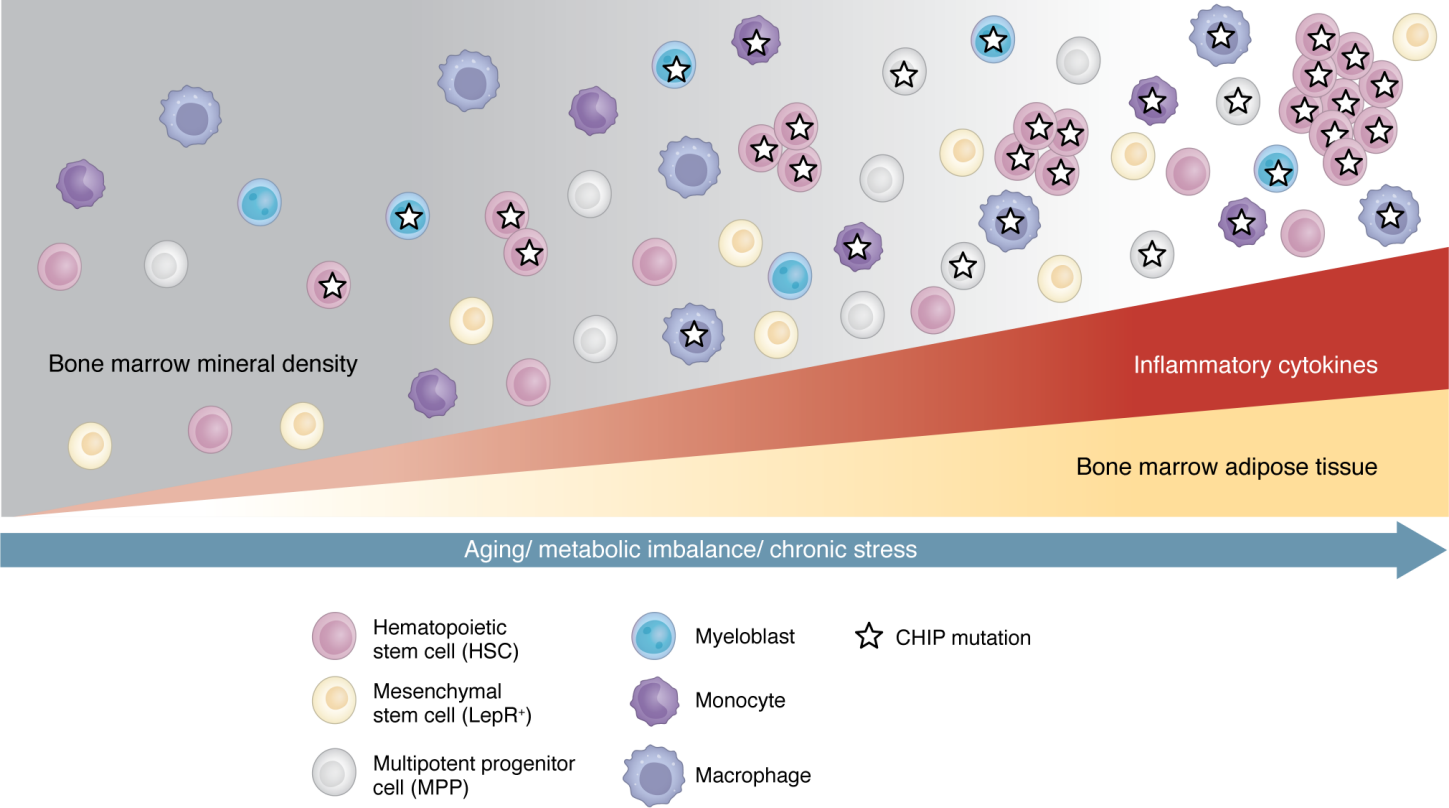
Changes to the BM niche with aging, metabolic imbalance, and/or chronic stress can promote CHIP. The BM niche is composed of multiple cell types that support long-term HSC function. Aging, metabolic imbalance, and chronic stress can result in changes to the BM niche that impair HSPC fitness in a way that could lead to the selection for mutant HSPCs and the eventual clonal outgrowth of mature blood cells associated with CHIP. This includes changes to the MSCs and increased inflammation and adipose tissue, together with reduced BM mineral density. Other changes with aging that are not depicted here include the loss of osteolineage cells, reduced innate and adaptive immunity, and phenotypic changes in HSPCs and their more mature progeny.

The hematopoietic niche was initially defined in 1978 as “the stem cell in association with other cells which determine its behavior” (16). Since then, decades of research have identified critical functions and cellular constituents of the BM niche that regulate hematopoiesis. The adult BM niche is predominantly localized to trabecular bone and is composed of a variety of cell types that support long-term hematopoietic stem cell (HSC) function, including endothelial cells, mesenchymal stem/stromal cells (MSC), osteolineage cells, and adipocytes (Figure 1). Many hematopoietic populations also contribute to HSC maintenance, including megakaryocytes, lymphocytes, and macrophages. The BM is highly vascularized and innervated with sympathetic and parasympathetic nerve fibers, providing a conduit of cytokine, chemokine, and endocrine signaling that regulates HSC activity in response to homeostatic cellular turnover, circadian cues, acute physiological needs for hematopoietic replenishment, and immunological “danger” signals associated with host defense and tissue-repair mechanisms (17–19). HSCs within the adult BM niche are primarily housed in perivascular beds (20), which can be subdivided into sinusoidal and arteriolar regions (21, 22). This spatial organization may be important in regulating quiescence versus cell-cycle activation in HSC, as sinusoidal perivascular beds could provide more exposure to nutrients, cytokines, and oxygen to support HSC cell-cycle activity (21, 22). However, others have shown quiescent stem cells localized proximal to sinusoids (23). While the exact structure of the BM niche and the organization of HSCs within these structures remains a matter of debate, many key cellular components have been identified based on cell type–specific gene knockouts and functional studies. For instance, HSCs interact extensively with leptin receptor–expressing (LepR^+^) MSCs, which promote HSC localization and maintenance in the BM through production of CXCL12 and stem cell factor (SCF). The complete array and role(s) of BM niche cell types is a topic extensively reviewed elsewhere (24, 25).

Dysregulation of the cellular composition and/or biophysical properties of the niche, due to systemic changes such as aging, chronic inflammation, metabolic imbalance, or environmental exposure, can affect the functional properties of HSCs, thereby establishing conditions that may promote clonal selection. This Review will summarize our current understanding of how these factors directly affect CHIP development and progression, with key studies summarized in Table 1. We will integrate these studies with the spectrum of lifestyle factors and CHIP-associated disease risks, highlighting current gaps in knowledge and opportunities for further study.

## CHIP and hematopoietic development: where does the journey begin?

When CHIP clones emerge remains a critical open question. Like Homer’s epic *Odyssey*, our understanding of CHIP begins in medias res — somewhere in the middle of the chain of events. Sequencing studies have identified rare HSPC clones harboring CHIP mutations in most individuals aged 50–60, with longitudinal stability and presence in multiple hematopoietic lineages. These data suggest that initiation of mutant clones occurs much earlier in life (26). Indeed, it is reasonable to assume that any given mutation occurs with similar probability throughout life in concordance with physiological rates of mutation accumulation. Hence, while CHIP is associated with aging, mutant HSPC clones may trace their origins to a much earlier point in hematopoietic development, potentially during fetal life. Although the topic is more comprehensively reviewed elsewhere (17, 27), we will briefly summarize our current understanding of hematopoietic development to provide context for addressing current gaps in knowledge. In mammals, definitive HSCs (defined as functionally capable of reconstituting the entire hematopoietic system) first arise during fetal development from a region of the embryonic mesoderm called the aorta-gonad-mesonephros (AGM) (28). In parallel with definitive HSCs, a transient population of fetal HSCs (termed developmentally restricted HSCs [drHSCs]) and an embryonic multipotent progenitor (eMPP) population arise, which are responsible for early life blood production, limited adult multilineage output, and lifelong lymphoid output (29, 30). Contrary to previous dogma, definitive HSC expansion occurs after BM colonization, while drHSC/eMPP expansion occurs in the fetal liver (29, 31). BM colonization is accompanied by BM ossification and vascularization, thereby giving rise to the foundational BM niche (17, 32). Concomitant with phases of HSC expansion, some evidence suggests that mutations can arise during fetal development. By reconstructing hematopoietic phylogenies using whole-genome sequencing of 12 MPN patients, one study reported *DNMT3A* and *JAK2^V617F^* mutations arising as early as 8 and 33 weeks of gestation, respectively (33). Moreover, a retrospective cohort study of the UK Biobank reported an association between abnormal birth weight, both low and high, and CHIP incidence, with a particular association (OR, 1.04 per 1 kg increase) between *DNMT3A* CHIP and higher birth weight (34). Although these studies showcase great strides in improved detection of rare mutant HSPC clones that allow us to infer potential origins for CHIP-associated mutations, more work is required to precisely identify when CHIP mutations, and the processes that select for them, occur in an individual’s life.

## Genotoxins and inflammation: the BM’s Scylla and Charybdis

Much like the two sea monsters Odysseus must navigate between, the BM must navigate cellular stresses that can severely impair hematopoietic function and potentially select for mutant HSPCs (35). We will begin our discussion with the well-characterized contributions of genotoxic agents, such as chemotherapeutics and other factors influencing mutation-driven clonal expansions in HSPC pools.

### Cytotoxic agents.

Cytotoxic agents, such as chemotherapeutics and radiation, are commonly employed to (somewhat) selectively eliminate malignant cells by eliciting DNA damage or impairing key cellular functions, such as DNA replication, transcription, and damage repair, or by inducing metabolic stress (36). Cytotoxic drugs are also employed as myeloablative agents in the setting of HSC transplantation (36, 37). In either case, cytotoxic agents are often inciting stimuli for expansion of HSPCs harboring mutations in DDR genes, such as in *TP53* and *PPM1D,* which confer resistance to DNA-damaging insults (38–40). Moreover, cytotoxic drugs may affect the niche in ways that promote MSC and HSC senescence (41, 42). *TP53* mutations are most highly associated with therapy-related MPNs (t-MN) and treatment-induced clonal hematopoiesis (t-CH) (43–45). *TP53* mutations also contribute to increased risk of heart failure in cancer survivors (46). Loss of *Trp53* in mouse HSPCs promotes maintenance of stem cell potential and fitness following γ irradiation (47–49), leading to potent selection for *Trp53*-mutant HSPCs. Although the potential mechanisms underlying *TP53*-mutant HSPC expansion have not been fully clarified, p53 plays important roles in regulating HSC quiescence, self-renewal, and proliferation (47, 49–51).

*TP53* mutations account for 40% of t-MN cases (44), likely due to t-MN’s association with a myriad of cytotoxic agents (38), while *PPM1D* gain-of-function mutations account for roughly 20% of non–*TP53*-mutant t-MN cases (52) and are driven primarily by platinum-based chemotherapies (38, 52–54). Interestingly, *PPM1D-*mutant HSPCs harbor gain-of-function truncating mutations at exon 6 that induce constitutive PPM1D protein expression, desensitizing these mutant HSPCs to DDR-induced apoptosis (52–54). Crosstalk occurs between PPM1D and p53, in which *PPM1D* is transcriptionally upregulated by p53. PPM1D in turn suppresses various DDR proteins, including p53 (55, 56). A recent murine study revealed the importance of this synergy in myeloid malignancy, as resistance to PPM1D inhibitors is driven by p53 inactivation (57). Moreover, genetic ablation of *Ppm1d* reduced HSC competition and serial replating capacity, suggesting its involvement in promoting HSC fitness (57). While its role in normal HSCs could portend toxicity issues, ablating *Ppm1d* sensitized *MLL-AF9*–expressing leukemia cells to cisplatin treatment, suggesting its therapeutic relevance in myeloid malignancy (57). The role of *Ppm1d* mutations in AML development was further corroborated in vivo. While a single dose of γ radiation or two doses of 5-fluorouracil (5-FU) delivered over two weeks failed to initiate leukemia development in *Ppm1d* mutant mice, sequential treatment with four doses of γ radiation over eight weeks induced eventual AML transformation in a proportion of *Ppm1d*-mutant mice (58). These data suggest that malignant transformation depends on cumulative exposures to genotoxic stressors. Furthermore, chemotherapeutic agents, specifically those with neurotoxic properties such as cisplatin, can damage the sympathetic nervous system in the BM, impairing regeneration of both HSPCs and stromal constituents of the niche. In this setting, induction of nerve growth factor or deletion of *Tp53* in sympathetic nerves rescued BM regeneration after a seven-week course of cisplatin (59). One may speculate that mutations in *Ppm1d* or *Tp53* could reduce the dependence of HSPCs on BM sympathetic innervations for their regenerative potential, thereby allowing them to outcompete normal HSPCs during hematopoietic recovery in a chemotherapy-damaged BM niche. Collectively, these studies show that chemotherapeutic agents constitute a potent mechanism for mutant HSPC selection, with impaired BM niche function as a potential contributing factor.

### Inflammatory signaling.

Inflammation is a complex biological response to harmful stimuli, e.g., pathogens or tissue damage, that involves a coordinated series of events including cytokine/chemokine signaling, activation and recruitment of innate immune cells, and ideally, resolution of the inciting stimulus (60, 61). While inflammation is a critical defense mechanism against infection and injury, dysregulated and/or chronic inflammation can contribute to various disease pathogeneses. Thus, a key area of interest is understanding how acute and chronic inflammation affect HSPC proliferation, differentiation, and self-renewal in normal and malignant hematopoiesis. Although this topic has been comprehensively reviewed elsewhere (62–65), several recent studies have considerably advanced our understanding of the role of inflammation in promoting CHIP initiation or progression, while also uncovering new questions.

Previous work revealed synergy between inflammation and selective expansion of certain mutant HSPC clones. Notably, *Tet2*-mutant HSPCs were shown to selectively expand in response to TNF-α (in vitro) and LPS (in vivo, for four weeks) stimulation (66, 67). This activity has been mechanistically linked to the STAT3/IL-6 signaling axis (67, 68). Moreover, recent work has reinforced IL-1 signaling as a driver of *Tet2*-deficient CHIP (69–71). IL-1 signaling is necessary and sufficient for *Tet2-*deficient clonal expansion, as stimulating mice with IL-1α or IL-1β for at least two weeks accelerated *Tet2*-mutant HSPC expansion, while genetic ablation or pharmacologic inhibition of the IL-1 receptor normalized *Tet2*-mutant HSPC expansion (69, 70). Mechanistically, *Tet2*-deficient HSPCs resisted IL-1–mediated depletion via DNA hypermethylation at prodifferentiation enhancer regions, which epigenetically primed them to favor self-renewal. While IL-1β stimulus promoted demethylation at these enhancer regions in WT HSPCs, resulting in myeloproliferative gene upregulation and subsequent differentiation, *Tet2*-deficient HSPCs maintained sufficient methylation at these regions to prevent their differentiation, thus mediating their expansion over their WT counterparts under chronic inflammatory stress (70), mirroring previous work demonstrating IL-1β–mediated selection for HSPCs with *Cebpa* loss (72).

While HSPC competitiveness is at least partially defined by how well mutant HSPCs resist chronic stress–induced BM depletion and terminal differentiation (73), mutation-specific phenotypes also contribute to a competitive advantage. Previous work comparing *Dnmt3a*- and *Tet2*-knockout models revealed that *Tet2*-deficient HSCs exhausted at the same rate as their WT counterparts, but loss of *Dnmt3a* effectively immortalized HSCs (74, 75). Other studies showed that loss of *Tet2* favored myeloid lineage bias in HSPCs (69, 70), suggesting a divergent model in which *Dnmt3a*-deficient CHIP is primarily driven by HSCs, while *Tet2*-deficient CHIP is driven primarily by multipotent progenitors (74). Similarly, mutant HSPC competitiveness may also depend on the inflammatory stimulus. While *Tet2*-deficient HSPCs preferentially expand in response to LPS and IL-1, as discussed above (66, 67, 69–71), *Dnmt3a*-knockout HSPCs expand under chronic (four weeks) IFN-γ stimulation in vivo and, to a lesser extent, IL-1β stimulation (76, 77). On the other hand, four weeks of stimulation with LPS, TNF-α, and poly I:poly C (pIpC) failed to promote selective expansion of *Dnmt3a*-knockout HSCs in this model (76, 77). In contrast, endogenous TNF-α signaling facilitated selective expansion of HSPCs carrying a heterozygous *Dnmt3a^R878H^* mutation (which recapitulates the human *DNMT3A^R882H^* mutation), suggesting potential differences in response to TNF-α stimulation between genetic knockouts and point mutations in *Dnmt3a*. (76, 77). In this latter study, the aging-associated fitness advantage of *Dnmt3a*-deficient HSCs required TNF-α/TNFR1 signaling, loss of which normalized HSC fitness without affecting lineage output. However, genetically ablating TNFR2 resulted in myeloid lineage bias without affecting HSC fitness (77). While TNFR signaling has not been studied in vivo in *Tet2*-deficient mouse models, in vitro data suggest that loss of *Tet2* and *Dnmt3a* both endow HSPCs with enhanced survival and/or clonogenic activity in response to TNF-α signaling (66, 67). Whether differential expression and/or signaling via TNFR1 or -2 underlies the differences in self-renewal activity between *Tet2*- and *Dnmt3a-*deficient HSCs remains an open question.

**Figure 2:**
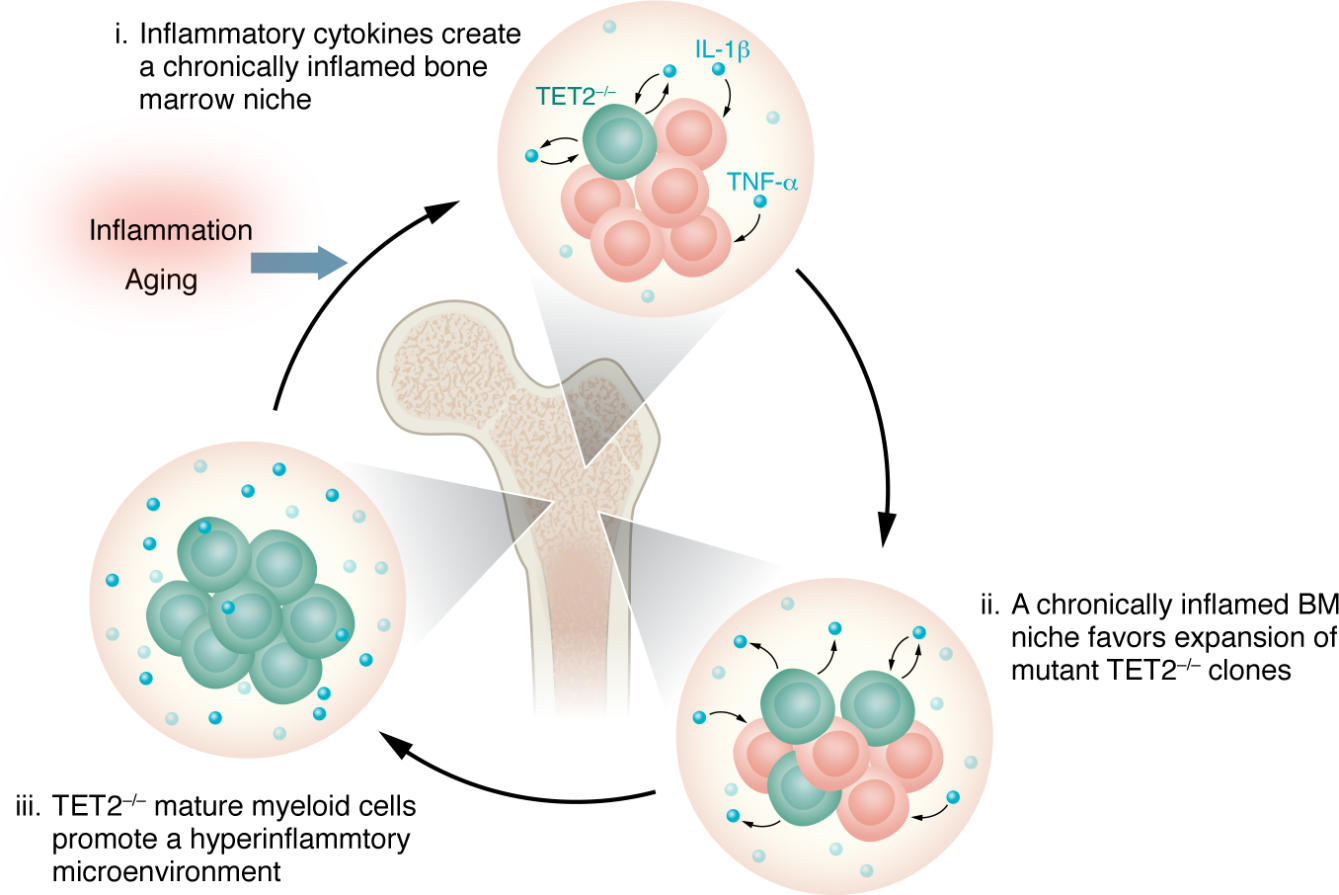
A model of an inflammatory feed-forward cycle in CHIP. *Tet2* deficiency leads to myeloid skewing and an increase in the number of mature myeloid cells carrying the mutation, in the BM and elsewhere. Because TET2 regulates inflammatory gene expression, its loss in myeloid cells leads to a hyperinflammatory BM niche, driven by elevated IL-1 and TNF-α signaling. This, in turn, has been shown to further promote *Tet2^–/–^* clonal expansion. Thus, *Tet2*-mutant HSPCs may establish conditions in the BM that favor their own expansion, effectively creating a feed-forward loop. This model may be relevant to other common CHIP mutations that demonstrate selective advantages under inflammatory stress.

Finally, these recent studies provide rationale for an interesting hypothesis related to *Tet2*-mutant clonal selection. Loss of *Tet2* promotes myeloid skewing (78), increasing peripheral mature myeloid cells, such as macrophages and neutrophils (79), that similarly carry this mutation. As *Tet2* regulates inflammatory gene expression (80), its loss in myeloid cells promotes a hyperinflammatory phenotype that can further shape the hematopoietic niche, impairing normal hematopoiesis (81, 82). This hyperinflammation also promoted *Tet2*-deficient clonal expansion via IL-1 (69, 70) and TNF-α signaling (66), thus creating an effective feed-forward loop (Figure 2) by which *Tet2*-deficient myeloid cells shape a niche that favors *Tet2*-deficient HSPC expansion (69, 70, 83). The extent to which such myeloid:HSC feed-forward loops drive selection in other CHIP mutant contexts remains largely an open question.

Beyond epigenetic modifiers, HSPCs harboring the *JAK2^V617F^* mutation also preferentially expand in BM under inflammatory conditions. Previous work established that *JAK2^V617F^* HSPCs expand at the expense of HSC self-renewal (84), and other work shows that treating mice harboring *JAK2^V617F^* HSPCs with IFN-γ, TNF-α, and/or TGF-β enhances *JAK2^V617F^* HSPC expansion in a DUSP1-dependent manner (85). Recent work also revealed a role for IL-1β in promoting MPN disease initiation in *JAK2^V617F^* HSCs by enhancing their early expansion in the BM (86), mirroring a previous study in which *JAK2^V617F^* hematopoietic cells exhibited increased IL-1β secretion, contributing to sympathetic nerve damage and subsequent MSC attrition in the BM niche (86, 87). Indeed, a chronic inflammatory state is a key feature of many MPNs (88), which are often characterized by clonal expansion of HSPCs with gain-of-function mutations in JAK2/STAT3/STAT5 pathways (89). However, it is unclear whether increased IL-1β secretion of *JAK2^V617F^* hematopoietic cells directly influences the dynamics of parental HSCs through paracrine or autocrine signaling (90).

Collectively, these studies suggest that inflammation plays a critical role in establishing conditions in the BM niche that select for mutant HSPCs through a combination of cell-intrinsic and -extrinsic mechanisms. However, while these studies conceptually illustrate the role of inflammation as a selection mechanism, caveats and limitations remain to be addressed. The extent to which treatment with single inflammatory mediators recapitulates selection for CHIP mutations is not clear, as physiological inflammation typically involves multiple inflammatory signals. Likewise, few studies have provided direct comparisons between inflammatory factors to assess which ones play a greater or lesser role in selecting for specific mutations. Coordinated studies that facilitate direct comparisons of inflammatory mediators, combined with the use of aging and chronic disease models in mice with targeted knockouts of key inflammatory pathway components, may offer an opportunity to clarify the role(s) of individual factors.

Finally, direct evidence for the role of inflammatory signaling as a driver of CHIP in humans remains relatively limited, though its roles in CHIP-related morbidities such as cardiovascular disease (CVD) are more clearly delineated in clinical studies (91). Analysis for CHIP mutations in the Canakinumab Anti-inflammatory Thrombosis Outcomes Study (CANTOS) clinical trial cohort (ClinicalTrials.gov NCT01327846), in which canakinumab (anti–IL-1β antibody) was administered to patients with prior myocardial infarction, revealed decreased subsequent major adverse cardiovascular events (MACEs) in subjects with *TET2* mutations relative to those without CHIP (91). Furthermore, new clinical trials are investigating the efficacy of canakinumab in preventing evolution to malignancy in individuals with clonal cytopenia of undetermined significance (CCUS) (NCT05641831) as well as MDS (NCT04239157). Recent CANTOS data have also shown improvement in anemia and inflammatory marker levels following canakinumab treatment among individuals with CHIP (92). Multiple biologics and antibodies that block cytokines such as TNF-α and IL-6 are in clinical use, typically for rheumatoid diseases (93, 94). These could be efficacious in limiting further selection for specific CHIP mutations and/or CHIP-associated diseases, though safety concerns associated with risk for severe infection have been previously noted (95).

## Psychosocial stressors: the Sirens’ wail

Odysseus chose to hear the Sirens’ song while lashed to his ship’s mast. However, psychosocial stressors are a generally unavoidable feature of modern society and include stresses associated with interpersonal conflict and social competition as well as job-related stressors related to achievement of goals and real or perceived impacts on social status (96). Although psychosocial stress responses (akin to the “fight or flight” response) likely evolved to prepare the body to respond to immediate threats of injury, persistent activation of these responses can become maladaptive and negatively affect health (97). However, the impact of psychosocial stress on the hematopoietic niche and its relationship to CHIP remain unknown, despite links to CHIP-associated diseases such as CVD (98). Notably, a study using a mouse model of intruder-mediated social defeat reveals HSPC mobilization from the BM and increased extramedullary monocyte, neutrophil, and erythrocyte production up to 24 days after stress cessation (99). However, whether these hematopoietic changes promote CHIP is unknown and may represent a critical area for future study given the relative ubiquity of social stressors in modern society. Moreover, while long-duration exposure to night-shift work, which disrupts the circadian biological clock (100), is inconclusively linked to hematopoietic cancers (101), its link to nonmalignant CHIP-associated morbidities such as CVD is more evident (102). Below, we offer rationales for investigating potential mechanisms that link impacts on HSPCs caused by circadian rhythm disruption with somatic evolution in the BM, leading to CHIP and its sequelae.

Circadian rhythms, which are the body’s internal clock, regulate physiological processes, including sleep-wake patterns, hormone regulation, body temperature, and systemic metabolism over an approximately 24-hour period. HSPC proliferation dynamics are dictated by circadian rhythms through sympathetic innervations that regulate hematopoietic and nonhematopoietic cells within the BM niche (103). These sympathetic innervations influence overlapping cycles of HSPC proliferation and DNA synthesis (104), circadian changes in CXCL12 secretion from the niche that regulate HSPC BM egress (105, 106), and rhythmic changes to vascular permeability driven by noradrenaline release in concert with bipotent bursts of TNF-α (107). Interestingly, recent work revealed that sleep interruption exerts lasting epigenetic changes that skew HSPCs toward myeloid lineage bias (108). Similarly, a separate study, which developed a novel murine model of prolonged sleep deprivation (up to 4 days), revealed increased accumulation of neutrophils and multiple proinflammatory cytokines in the peripheral blood, including IL-6, IL-17, and TNF-α, within 24 hours of sleep deprivation (109). Finally, a longitudinal study of 130,343 women aged 50–79 years found that those with higher sleep disturbances were at 22% higher risk of leukemia, particularly myeloid leukemia (110). Altogether, circadian rhythm disruption can profoundly alter hematopoietic homeostasis via systemic increases in proinflammatory cytokines, reduction of key chemokine cues that regulate neutrophil-recycling processes, and lasting epigenetic changes in HSPCs (108, 109, 111) that could select for HSCs with CHIP mutations. Intriguingly, a recent murine study utilized sleep fragmentation (112) throughout an 80-day competitive transplant experiment between WT and *Tet2-*deficient BM. This work showed that sleep disruption accelerates *Tet2*-mutant cell expansion in peripheral blood (113). However, more work using these novel paradigms is necessary to directly investigate how circadian rhythm disruption influences CHIP.

## Diet and exercise: whither the Lotus-Eaters?

While the vegetarian diet on the island of the Lotus-Eaters would presumedly be healthy, the direct impact of diet on CHIP development or progression is largely unknown. A recent retrospective cohort study of 44,111 participants (mean age = 56.3 years) from the UK Biobank reported that CHIP was present in 162 of 2,271 (7.1%) participants with an unhealthy diet, 2,177 of 38,552 (5.7%) participants with an intermediate diet, and 168 of 3,288 (5.1%) participants with a healthy diet; dietary metrics were scored by weekly frequency of intake of fruits and vegetables versus processed and unprocessed meat. While no statistically significant differences were observed between the distribution of CHIP mutations across dietary classes, the majority (58.6%) of participants with CHIP that had an unhealthy diet had *DNMT3A* mutations, while 16% of participants had *TET2* mutations and 6.2% had *ASXL1* mutations (114). Direct observations using murine models of *Tet2*-deficient CHIP and an obesogenic diet (high fat and sucrose content) showed clonal expansion similar to that seen with aging (13); however, these experiments did not include direct comparisons of CHIP mutation–bearing mice between regular and obesogenic diets. Interestingly, diabetes-induced hyperglycemic stress can progress *Tet2* CHIP to a full-blown MPN in mice (68). Congruent with other studies, poor diet and obesity may exacerbate CHIP progression through increased inflammatory signaling (13, 68, 115). While others have established inflammation as a driver of mutant HSPC expansion (66, 69, 71, 76, 77, 85–87, 116), these studies also implicate the sufficiency of an obesity-induced inflammatory state in promoting mutant HSPC clonal expansion.

Indeed, previous studies have linked poor diet to systemic inflammation (117). Of note, a high-fat diet increases gut permeability and subsequently upregulates expression of proinflammatory cytokines (118, 119). Conversely, dietary restriction can normalize some aging-associated HSC phenotypes, such as increased leukocyte number, myeloid lineage bias, and decreased quiescence due to suppression of IGF1 and IL-6 signaling (120, 121). Similarly, short-term fasting and refeeding induced autophagy in HSCs, which in turn normalized glucose uptake and glycolytic flux of aged HSCs, improving their regenerative potential (122). These studies point to an exciting unanswered question in the field: can CHIP be mitigated by dietary intervention? Inferential evidence from an analysis of 8,709 women with a mean age of 66.5 years found that, while diet quality and physical activity had no association with CHIP prevalence, maintaining a normal BMI (18.5–25 kg/m^2^) was strongly associated with lower CHIP prevalence (123). Moreover, dietary restriction increased CXCL12/CXCR4 activity (124), which mitigates age-related HSC dysfunction and maintains normal ROS levels (125–127), constituting a potential avenue for mitigating CHIP clone expansion.

### Exercise.

In addition to dietary habits, regular exercise is crucial to maintaining a healthy body weight and fosters myriad other benefits (128, 129). However, whether exercise can mitigate CHIP clone expansion has not been investigated beyond an ongoing clinical trial (NCT03996239) studying the impact of aerobic exercise on CHIP progression. Of note, while the capacity of exercise to reduce adipose tissue deposits in humans is well known (130), a murine study showed that aerobic exercise can also decrease dietary-induced BM adipose tissue accumulation (131). Aging and obesity increased BM adipose tissue, which altered HSC polarization, diminished granulocyte and erythroid differentiation (132), reduced bone density, and profoundly remodeled the hematopoietic niche by altering expression of extracellular matrix signaling pathways associated with proliferation and differentiation (133). In this mouse study, a 12-week treadmill regimen delayed high-fat diet–induced obesity, increased trabecular bone mass, and improved the BM microenvironment by partially inhibiting adipokine signaling (131). Moreover, exercise diminished adipocyte leptin production, which in turn augmented production of quiescence-promoting factors secreted from LepR^+^ MSCs in the BM. Notably, while exercise in this study reduced chronic leukocytosis, emergency myelopoiesis was not compromised (131). Moreover, exercise-trained mice had higher BM reconstitution after irradiated transplant even when fed a high-fat diet, suggestive of improved BM niche function. Thus, while exercise could mitigate CHIP through balancing the inflammatory milieu and partially correcting the adverse composition of the damaged niche, such benefits have not been evaluated in the setting of CHIP, leaving a critical open question in the field.

## Aging: the end or beginning of the odyssey?

While Homer’s protagonist spent a decade escaping many perils, he did not escape the passage of time. Likewise, the only truly inescapable alteration to the niche, and the most dominant determinant of who develops CHIP, is aging. Our species’ evolution has favored tissue maintenance to maximize reproductive output. As this maintenance wanes in postreproductive periods, so does the integrity of the HSC niche, and in combination with a lifetime of insults, comes the inevitable decline in our soma associated with old age (134). It is possible to mitigate these insults by maintaining proper diet, sleep, and exercise routines, reducing chronic stress, and managing inflammatory states. However, aging poses a unique challenge for the BM niche that incorporates many features of the insults outlined above. It is associated with (a) lower bone mineral density, (b) stiffening of the BM matrix, (c) increased inflammation (termed “inflammaging”), and (d) various changes in MSCs (135). These changes can collaborate with cell-intrinsic features of HSC aging (136–138), including proliferative attrition and replicative stress, to profoundly impair hematopoietic fitness and promote mutant clonal selection (Figure 1).

Reduced bone mineral density of older mice diminishes MSC proliferation and response to osteogenic growth factors (139), resulting in decreased niche factors such as Netrin 1 (140) and POT1A (141), leading in turn to DDR defects, BM ROS, and adipose tissue accumulation (141), which can alter HSC polarization, differentiation (132), and long-term function (133). Furthermore, aging increases central marrow LepR^+^ MSC, which, in addition to having a deleterious effect on the sinusoidal vasculature, is associated with decreased osteoprogenitor function (142). The resultant degraded and inflamed BM niche may parallel developmental HSC/eMPP extinguishment (29, 30), which suggests a potential concomitance between myeloid-skewing extrinsic cues from the niche and lymphoid attrition with age.

In contrast to CHIP’s low prevalence, defined by a 2% variant allele frequency threshold in nonelderly, rare clones harboring CHIP mutations are present in most people before age 70 when analyzed by error-corrected sequencing methods (26). This suggests that before old age, most CHIP clones either do not expand or expand very slowly, and the altered BM microenvironment associated with aging might be responsible for the emergence of CHIP at clinically relevant rates.

However, there is a paucity of studies directly investigating the aging-specific changes that contribute to CHIP clone selection in humans. Mouse models provide some evidence that aging, primarily through inflammatory signaling, favors certain mutant HSCs. Indeed, age-associated “inflammaging” (143, 144) can drive rapid myelopoiesis and IL-1β production, impairing BM niche function (142). A recent mouse study showed that *Dnmt3a^R878H^* BM cells expanded faster when transplanted into older recipient mice, due to their reduced sensitivity to LPS- and TNF-induced necroptosis in the aged setting (145). Recent work revealed increased expansion of *Tet2*-deficient HSPCs starting 7 months after selective, inducible *Tet2* deletion. Intriguingly, IL-1α correlated with the rate of expansion, and deleting the IL-1 receptor in *Tet2*-deficient HSPCs prevented their expansion (69). Additionally, gut microbiome changes in aged mice were associated with increased IL-1α and IL-1β in the BM, reducing engraftment potential and increasing myeloid bias of HSCs (146). As IL-1β promotes expansion of *Tet2*-deficient HSPCs (70), an aged microbiome may promote *Tet2*-deficient HSPC expansion; however, this hypothesis remains untested. In addition to these cell-extrinsic cues, several cell-intrinsic changes have been described in aged HSCs (147). Aged HSCs showed altered heterochromatin landscapes associated with increased aberrant expression of repetitive elements, such as endogenous retroviral sequences that induce interferon-regulated genes. Deleting *Tet2* in aged cells partially mitigated these heterochromatin changes, leading to a more youthful transcriptomic profile. Moreover, reverse transcriptase inhibitors reduced the amount of cytosolic DNA in old WT HSCs, rescuing their fitness compared with old *Tet2*-deficient HSCs in vitro, although whether this approach can abrogate *Tet2*-deficient HSPC expansion in vivo is unknown (83). Notably, others have suggested implementing reverse transcriptase inhibitors as rejuvenating treatments to correct aberrant expression of repetitive elements as well (148).

Finally, impaired glucose metabolism (122) and hyperglycemia are known features of inflammaging, and introduction of *Tet2* mutations into hyperglycemic *Ins2^Akita/+^* mice triggered age-dependent increases in mortality, hyperactive inflammation, and progression to an MPN/AML phenotype (68), demonstrating key links between inflammation and aging-associated metabolic phenotypes that may promote CHIP. Moreover, the preferential expansion of mutant *Asxl1* HSCs in aged mice also relied on a cell-intrinsic mechanism involving Akt/mTOR activation (149), which can regulate multiple cellular metabolic pathways (150). However, as in the case of *Tet2*-deficient HSPC expansion described above (69), selection was only evident several months after induction. Furthermore, this process may be partially driven by attenuated responses to inflammatory factors in *Asxl1* mutant HSPCs, as shown in zebrafish models (151). In addition to aging-dependent cell-intrinsic and -extrinsic changes, mutant clone evolution due to secondary genetic and epigenetic events could augment their fitness. *Tet2*-deficient differentiated cells expressed higher levels of inflammatory cytokines, such as IL-6 (152) and IL-1 (153, 154), suggesting that *Tet2*-mutant cells can alter the microenvironment to fuel their own expansion in a positive feedback loop (Figure 2). Disentangling these effects from those derived from aging will require careful experimental design.

## Conclusions and discussion

Here we have consolidated seminal and recent works that shape our current understanding of CHIP development and progression as a product of cell competition, in which changes in the BM niche dictate genotype-dependent HSC fitness (155). However, as we have alluded to throughout our discussion, much work is still needed to rigorously characterize driving factors of CHIP.

**Figure 3:**
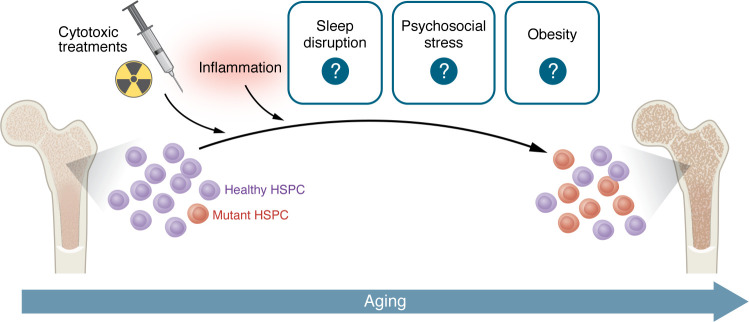
Summary. Aging is a known driver of CHIP. Recent work has further cemented our mechanistic understanding of genotoxic insults and inflammation as drivers of mutant HSPC expansion. However, how lifestyle factors, such as circadian rhythm disruption, psychosocial stress, and dietary/exercise habits, contribute to CHIP progression or development remains largely unknown. These factors constitute likely areas for future investigation, from both the standpoint of clinical intervention and from the basic science standpoint of establishing mechanistic understanding of somatic evolution of the BM and other tissues with age.

While novel analysis of MPN patient sequencing data reveals crucial insight into the potential origins of CHIP mutations (33), it is unknown which, if any, CHIP mutations occur during fetal development. However, as we and others (155) have pointed out, the occurrence of the mutation may matter less than the changing microenvironment that dictates clonal fitness. Many studies have elucidated key mechanisms driving mutant HSPC expansion, including cytotoxic stress, inflammation, and aging. Of note, recent murine studies have demonstrated inflammaging as a clear driver of *Tet2*-deficient HSPC expansion. These studies also paint an interesting picture, echoed among other CHIP models, in which mutant HSPCs that selectively expand under inflammatory conditions can promote an inflammatory state via overproduction of dysregulated, hyperinflammatory myeloid progeny (Figure 2). This feed-forward pattern may be a mechanistic basis for CHIP and/or its progression to malignancy in some individuals (156). Moreover, lifestyle factors, such as circadian rhythm disruption, poor diet, and lack of exercise, can have profound impacts on normal hematopoiesis (13, 68, 108, 109, 111, 115, 131, 157), with a common role for increasing systemic inflammation. However, there are few studies that directly investigate how social stress affects the hematopoietic system and no studies that have investigated social stressors as a driver of CHIP. While clinical data and current clinical trials have revealed novel associations between some lifestyle factors and CHIP incidence (110, 114, 123), whether and how these lifestyle factors contribute to CHIP remain unknown (Figure 3).

Most of the studies discussed here are limited to “paradigmatic” CHIP mutations such as *TET2* or *DNMT3A*, whereas mechanisms promoting clonal selection for other mutations remain somewhat understudied. For instance, mutations in the SFs *SF3B1*, *SRSF2*, and *U2AF1* perturb hematopoiesis via several mechanisms, including myeloid skewing, impairment of the DDR, and exacerbated inflammatory responses (158–160). However, the extent to which these mechanisms contribute to selection remains unknown. This lack of information creates potential hazards in drawing inductive conclusions from limited data sets regarding the mechanism(s) driving selection for different CHIP mutations. CHIP mutations are often segregated into low-risk (*DNMT3A*, *TET2*, *ASXL1*) and high-risk (*SRSF2*, *SF3B1*, *JAK2*, *RUNX1*, *IDH2*, *TP53*) groups, where risk is defined as having increased incidence of myeloid neoplasms (161). However, while *TET2* may be considered low risk with respect to myeloid malignancy, it is high risk in the context of atherosclerosis (14, 162). These data suggest there are likely unique courses of somatic evolution for each mutation, which in turn may exacerbate specific clinical risks. Thus, determining the extent to which a changing BM niche (Figure 1) or activity of inflammatory feed-forward loops (Figure 2) favors one mutation or another will require tailored studies. For many of these mutations, further investigation is needed to evaluate whether CHIP-promoting conditions differ from those that result in a downstream disease phenotype (MDS/AML/CVD).

Finally, although the studies reviewed here highlight the independent impacts of selective factors such as inflammation or altered BM niche composition and function, an integrated view of how various contexts (old age, lifestyles, and exposures) alter the BM niche to differentially influence the fate/fitness of mutant HSPCs that confer different risks for malignant progression will be key to designing interventions that limit these risks. Unraveling these interactions will provide a more nuanced understanding of the mechanisms governing the clonal odyssey in the BM niche across (potentially) a lifetime.

## Author contributions

WES, BH, JD, and EMP conceived the project. WES, BH, and MDD wrote the original draft of the manuscript. WES, BH, MDD, JD, and EMP reviewed and edited the manuscript. JD and EMP supervised the project. Order of co–first authors was determined by the senior role of WES in developing the manuscript outline.

## Figures and Tables

**Table 1:**
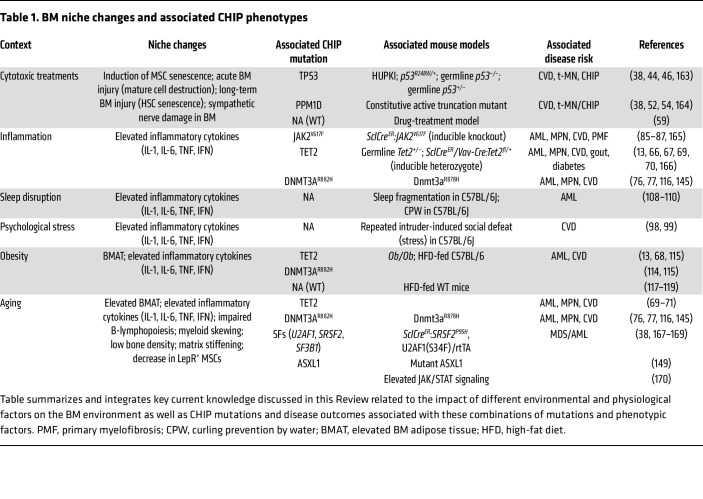
BM niche changes and associated CHIP phenotypes

## Version 1 08/01/2024

Electronic publication
